# Cross-Protective Immunity to *Leishmania amazonensis* is Mediated by CD4+ and CD8+ Epitopes of *Leishmania donovani* Nucleoside Hydrolase Terminal Domains

**DOI:** 10.3389/fimmu.2014.00189

**Published:** 2014-05-01

**Authors:** Dirlei Nico, Daniele Crespo Gomes, Marcus Vinícius Alves-Silva, Elisangela Oliveira Freitas, Alexandre Morrot, Diana Bahia, Marcos Palatnik, Mauricio M. Rodrigues, Clarisa B. Palatnik-de-Sousa

**Affiliations:** ^1^Laboratório de Biologia e Bioquímica de Leishmania, Departamento de Microbiologia Geral, Instituto de Microbiologia Paulo de Góes, Universidade Federal do Rio de Janeiro, Rio de Janeiro, Brazil; ^2^Laboratório de Imunologia, Instituto de Microbiologia Paulo de Góes, Universidade Federal do Rio de Janeiro, Rio de Janeiro, Brazil; ^3^Departamento de Microbiologia, Imunologia e Parasitologia, Escola Paulista de Medicina, Universidade Federal de São Paulo, São Paulo, Brazil; ^4^Departamento de Biologia Geral, Instituto de Ciências Biológicas, Universidade Federal de Minas Gerais, Belo Horizonte, Brazil; ^5^Programa de Pós Graduação em Clínica Médica Faculdade de Medicina, Universidade Federal do Rio de Janeiro, Rio de Janeiro, Brazil; ^6^Departamento de Microbiologia, Imunologia e Parasitologia, Centro de Terapia Celular e Molecular, Escola Paulista de Medicina, Universidade Federal de São Paulo, São Paulo, Brazil

**Keywords:** visceral leishmaniasis, cutaneous leishmaniasis, diffuse cutaneous leishmaniasis, cross-protection, prophylaxis, nucleoside hydrolases, recombinant vaccines

## Abstract

The nucleoside hydrolase (NH) of *Leishmania donovani* (NH36) is a phylogenetic marker of high homology among *Leishmania* parasites. In mice and dog vaccination, NH36 induces a CD4+ T cell-driven protective response against *Leishmania chagasi* infection directed against its C-terminal domain (F3). The C-terminal and N-terminal domain vaccines also decreased the footpad lesion caused by *Leishmania amazonensis*. We studied the basis of the crossed immune response using recombinant generated peptides covering the whole NH36 sequence and saponin for mice prophylaxis against *L. amazonensis*. The F1 (amino acids 1–103) and F3 peptide (amino acids 199–314) vaccines enhanced the IgG and IgG2a anti-NH36 antibodies to similar levels. The F3 vaccine induced the strongest DTH response, the highest proportions of NH36-specific CD4+ and CD8+ T cells after challenge and the highest expression of IFN-γ and TNF-α. The F1 vaccine, on the other hand, induced a weaker but significant DTH response and a mild enhancement of IFN-γ and TNF-α levels. The *in vivo* depletion with anti-CD4 or CD8 monoclonal antibodies disclosed that cross-protection against *L. amazonensis* infection was mediated by a CD4+ T cell response directed against the C-terminal domain (75% of reduction of the size of footpad lesion) followed by a CD8+ T cell response against the N-terminal domain of NH36 (57% of reduction of footpad lesions). Both vaccines were capable of inducing long-term cross-immunity. The amino acid sequence of NH36 showed 93% identity to the sequence of the NH A34480 of *L. amazonensis*, which also showed the presence of completely conserved predicted epitopes for CD4+ and CD8+ T cells in F1 domain, and of CD4+ epitopes differing by a single amino acid, in F1 and F3 domains. The identification of the C-terminal and N-terminal domains as the targets of the immune response to NH36 in the model of *L. amazonensis* infection represents a basis for the rationale development of a bivalent vaccine against leishmaniasis.

## Introduction

Leishmaniasis is considered a severe public health problem with 12 million people currently infected, 350 million at risk (1, 2), and 4 clinical syndromes due to different *Leishmania* species: cutaneous (CL) (3–5), diffuse (DCL) (3), mucocutaneous (MCL), and visceral (VL). A bivalent vaccine that could generate protective immunity to the agents of the visceral and cutaneous syndromes would be economic and useful for the control of leishmaniasis (6) in countries where both diseases are endemic. First, second, and third generation vaccines have been developed against leishmaniasis (7, 8). Among the vaccines tested in the field, most are crude parasite vaccines against CL, with or without adjuvants (9, 10) that induced a maximum of 50% vaccine efficacy (9). The recombinant Leish-111f vaccine, on the other hand, was useful in the immunotherapy and immunochemotherapy of patients with CL and MCL (8) and in prophylaxis (11) but not in the therapy of canine VL (12). No human vaccine is available against VL.

The Leishmune^®^ veterinary vaccine against canine VL (13–16) contributed to the reduction of the incidence of the human and canine diseases (17). Its main component is the nucleoside hydrolase (NH) of *Leishmania donovani* (NH36) (18, 19). NHs release purines and pyrimidines from imported nucleosides, allow the synthesis of parasite DNA and its replication (20, 21) and are mandatory at the early infection. NH36 is a powerful antigen (22), a marker of the *Leishmania* genus (23, 24), which shows high homology to the sequences of NHs of other *Leishmania* species (25, 26), being thus a good candidate for a cross-protective bivalent *Leishmania* vaccine.

NH36 protected mice from *L. donovani* infection (27) and was identified among *Leishmania major* exo-antigens (28). As a genetic vaccine, it induced a TH1 immune response mediated by IFN-γ-producing CD4+ T cells (29) effective in mice prophylaxis against VL (30) and CL (28–31) and in mice (32) and dog immunotherapy against VL (33) indicating its potential use against both leishmaniasis.

Three recombinant peptides of NH36 representing the amino acids 1–103 (F1, N-terminal domain), 104–198 (F2, central domain), and 199–314 (F3, C-terminal domain) respectively, were generated and used to vaccinate mice (34). Protection against *Leishmania chagasi* was related to the C-terminal domain and was mainly mediated by a CD4+ T cell-driven response with a lower contribution of CD8+ T cells (34). Preliminary results indicated that, on other hand, both the C- and N-terminal domains determined the reduction of the size of footpad lesions of mice challenged with *Leishmania amazonensis* (34).

In this investigation, we aimed to study the cross-immunity generated by the peptide domains of NH36 of *L. donovani* used for prophylactic vaccination of mice against *L. amazonensis*. In order to study the generation of the humoral and cellular immune responses responsible for and to identify in this way, the immunogenic domains of NH36, which should be included in a potential future bivalent vaccine against VL and CL. We identified that the cross-protective efficacy responsible for protection against *L. amazonensis* was related to epitopes for CD4+ T cells of the C-terminal and epitopes for CD8+ T cells of the N-terminal domains of the NH, NH36.

## Materials and Methods

### Ethical statements

All mouse studies followed the guidelines set by the National Institute of Health, USA, the EU Directive 2010/63/EU, and the Institutional Animal Care and Use Committee approved the animal protocols (Biophysics Institute-UFRJ, Brazil, and protocol IMPPG-007). All procedures and euthanasia were performed under CO_2_ anesthesia, and all efforts were made to minimize suffering.

### Recombinant peptides of the NH36 nucleoside hydrolase of *Leishmania donovani* and homology to NH of *Leishmania amazonensis*

NH36 is composed of 314 amino acids (EMBL, GenBank™, and DDJB data bases, access number AY007193). Three fragments of the NH36 antigen composed, respectively, of the amino acid sequences 1–103 (F1), 104–198 (F2), and 199–314 (F3) were cloned in the pET28b plasmid system (34) (Patent: INPI Brazil PI 1015788-3.PCT/BR2011/000411) and expressed in *Escherichia coli* Bl21DE3 cells and purified in a Ni-NTA column (Qiagen). The fractions containing highly purified recombinant protein were extensively dialyzed against PBS buffer and stored at −80°C. To improve protein expression, F2 was further cloned in the pET28a (34). For homology analysis, we used the sequence of *L. amazonensis* NH A34480 (Scaffold1680 15191–16135) (35). The sequence alignment was obtained using the BLASTP of the GenBank.

### Prophylactic immunization, parasite challenge by *L. amazonensis*, and assessment of protection

Eight-week-old female Balb/c mice were vaccinated three times with 100 μg of NH36, F1, F2, or F3 recombinant proteins and 100 μg of SIGMA saponin (NH36sap, F1sap, F2sap, and F3sap vaccines, respectively) at weekly intervals, by the sc route. At week 4, mice were challenged in the right hind footpad with 10^5^ *L. amazonensis* (PH 8 strain) metacyclic promastigotes (31), which had been isolated from hamsters and maintained in Schneider’s axenic media supplemented with 10% fetal calf serum for one passage. The infected footpad thicknesses were measured weekly with a Mitutoyo apparatus and the thickness values of the non-infected left footpads were subtracted from them at each measure. Seven days after immunization and 6 weeks after infection, sera were collected for the anti-NH36 antibody assays and the intradermal response against *L. amazonensis* lysate (IDR) was measured in the footpads. Mice were sacrificed 6 weeks after challenge by euthanasia with carbon dioxide. The cellular immunity was assessed by flow cytometry analysis (FACS analysis), intracellular staining (ICS) of splenocytes, and cytokine-ELISA assays of splenocyte supernatants. For the assessment of long-term immunity, mice received the same immunization protocol but were challenged 1 month after the last vaccine dose. In these animals, cross-protection was evaluated by monitoring the sizes of footpad lesions and by determination of the parasite load in lesions after euthanasia by a limiting dilution assay as modified from de Oliveira Cardoso et al. (36).

### Detection of antibodies

Antibodies were measured in sera using an ELISA assay against NH36 recombinant proteins as previously described (34). The ELISA assay used 2 μg of NH36 per well (50 μl of a 40 μg/ml antigen solution) and goat anti-mouse IgG (Sigma) or goat anti-mouse IgG1, IgG2a, IgG2b, IgG3, IgM, and IgA horseradish peroxidase conjugated antibodies (Southern, Biotechnology Associates, Birmingham, AL, USA) in a 1:1000 dilution in the blocking buffer. The reaction was developed with *O*-phenyldiamine (Sigma), interrupted with 1 N sulfuric acid, and monitored at 492 ηm. Each individual serum was analyzed in triplicate in double-blind tests. Positive and negative control sera were included in each test. Results were expressed as the mean of the absorbance values (492 ηm) of the 1/100 diluted sera of each animal.

### Analysis of the cellular immunity

#### Intradermal response to leishmanial antigen (IDR)

The intradermal response against *L. amazonensis* lysate (IDR) was measured in the footpads. Briefly, mice were injected intradermally, in the right front footpad, with 10^7^ freeze-thawed stationary phase *L. amazonensis* promastigotes in 0.1 ml sterile saline solution. The parasites were obtained as amastigotes aseptically removed from *L. amazonensis* (PH 8 strain) infected hamster footpad lesions, transformed, and cultured in Schneider’s axenic medium at 26°C until they reached the stationary phase of growth and were then disrupted by three consecutive freeze-and-thaw cycles using liquid Nitrogen. The footpad thicknesses were measured with a Mitutoyo apparatus, both before and at 0, 24, and 48 h after injection. Injecting each animal with 0.1 ml saline in the left front footpad served as control. At each measurement, the values of the saline control were subtracted from the reaction due to the *Leishmania* antigen.

#### Anti-NH36-specific T cell immunity

Spleens were aseptically removed and disrupted in NaCl saline solution (Sigma Co., USA) using a Petri dish and nylon mesh, suspended to 11 ml with lysis solution (NH_4_Cl 8.29 g/l, KHCO_3_ 1 g/l, and EDTA 37.2 mg/l) and further centrifuged at 400×*g* for 5 min at 4°C until total red blood cell removal. The pellet was further washed with saline solution by centrifugation, incubated with 3 ml RPMI supplemented with 10% fetal calf serum, 0.05 mM 2-mercaptoethanol and antibiotics (200 U/ml of penicillin and 200 μg/ml of streptomycin), counted in a hemocytometer chamber. For cytokine dosage, splenocytes were distributed in 96 well flat-bottomed plates (Nunc, Roskilde, Denmark) with each well containing 10^6^ cells in a final volume of 200 μl and incubated, in the presence or absence of 5 μg of recombinant NH36 for 3 days at 37°C under a 5% CO_2_ atmosphere. RPMI supplemented medium was added as negative control. After this period, supernatants were harvested, centrifuged at 14,000 rpm for 11 s, and further stored at −70°C until dosage. Secretion of IFN-γ, TNF-α, and IL-10 was evaluated in the supernatants by an ELISA assay, using the mouse IFN-γ, TNF-α, and IL-10 BD OptEIA ELISA Set II kits (BD Bioscience) according to the manufacturer’s instructions. Splenocytes, after *in vitro* incubation, were processed for immunostaining with anti-CD4 (clone GK1.5) or anti-CD8-FITC (clone 53-6.7) monoclonal antibodies (R&D systems Inc.) and analyzed by flow cytometry analysis (FACS analysis) in a FACScalibur apparatus. Cells were analyzed by flow cytometry in a Becton Dickinson FACScalibur apparatus. Data were analyzed using the Win MDI program.

#### *In vivo* depletion of CD4+ or CD8+ T cells

Mice were vaccinated with three doses of F1sap and F3sap at weekly intervals were challenged with 10^5^ *L. amazonensis* infective promastigotes, 10 days after complete vaccination. One week after complete vaccination and on week 6 after challenge, the IDR against *L. amazonensis* lysate was assayed. *In vivo* depletion was performed by treating groups of F1- and F3-vaccinated mice with GK1.5 or 53.6.7 rat IgG MAb on days 2, 4, and 6 before challenge and on day 14 after challenge. Mice were treated with 50 μl of ascitic fluid containing an approximate 5 mg/ml MAb concentration. Control mice received the F1sap or F3sap vaccines and 0.05 mL of rat serum ip, equivalent to 0.25 mg of IgG, or nude mice ascitic fluids containing 0.25 mg of anti-CD4+ and/or anti-CD8+ antibodies. As determined by FACS analyses, the efficacy of depletion of CD4+ or CD8+ spleen cells before challenge was of 99.94 or 96% in anti-CD4+ or anti-CD8+ treated mice, respectively. The efficacy of depletion treatment was monitored by the increase of the size of footpad lesions along the 6 weeks of experiment. In addition, the parasite load in lesions on week 6 was evaluated by a limiting dilution assay (36).

### Statistical analysis

Means were compared by Kruskall–Wallis and Mann–Whitney non-parametrical tests. For the levels of IFN-γ and TNF-α induced by the F1 vaccine, we also used the confidence interval (95% CI) (Analyze-it). Correlation coefficient analysis was determined on a Pearson bivariate, two tailed test of significance (GraphPad Prism 6). The values of *R*^2^, which represents the fraction of the total variance in *Y* that can be explained by the variation in *X*, were obtained using linear regression analysis (Analyze-it).

## Results

**Figure 1:**
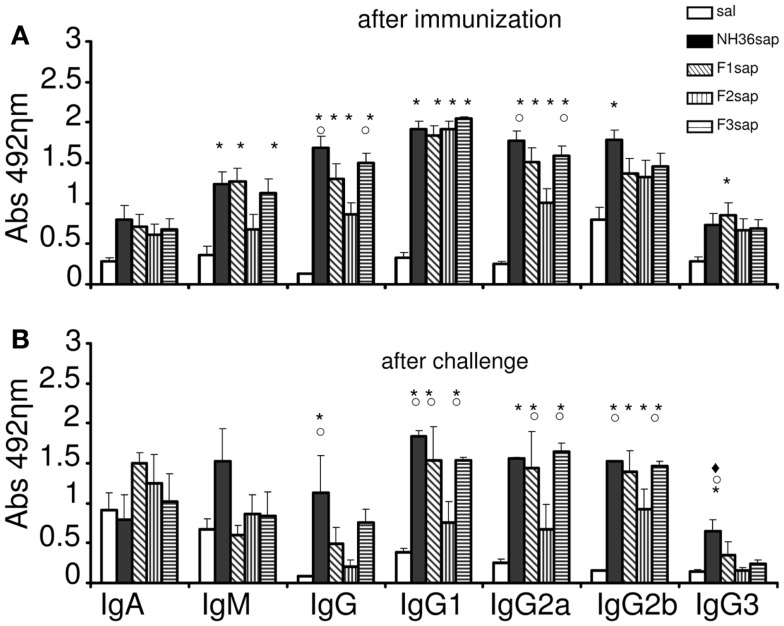
**Development of NH36-specific humoral immune response**. Bars represent the mean ± SE of the absorbance values of anti-NH36 antibodies from 1/100 diluted serum of three independent experiments after immunization (*n* = 3 mice per treatment in each experiment) **(A)** and two independent experiments after challenge (*n* = 7 mice per treatment in each experiment) **(B)**. *p < 0.05 from the saline control; ○*p* < 0.05 different from the F2sap vaccine; ◆*p* < 0.05 different from F3sap.

Mice were immunized with NH36, F1, F2, or F3 proteins and saponin, challenged with infective promastigotes of *L. amazonensis* at 4 weeks and euthanized 6 weeks after challenge. After immunization (Figure 1A), the humoral response against the NH36 antigen assayed by ELISA disclosed higher IgM, IgG, IgG1, and IgG2a antibody levels in the mice sera of all vaccines when compared to saline controls (*p* < 0.001). The F3sap vaccine showed the best performance, inducing IgG and IgG2a levels as high as NH36sap. Both the F3 and the F1 vaccines induced similar levels of IgM to the NH36 vaccine while the IgG2b was only enhanced by the NH36 and the IgG3 by the F1 vaccine, respectively (Figure 1A). After challenge, significant differences were observed among IgG, IgG1, IgG2a, IgG2b, and IgG3 antibodies (<0.001, for all antibody types) (Figure 1B). While the NH36sap vaccine showed the highest levels of IgG and IgG3 antibodies, the F3sap was as strong as the NH36 vaccine in the IgG1, IgG2a, IgG2b subtypes. Differently from what was seen before infection (Figure 1A), after challenge, the F1sap and F3sap vaccines showed levels of IgG1 and IgG2a antibodies significantly increased above the F2sap vaccine (Figure 1B).

**Figure 2:**
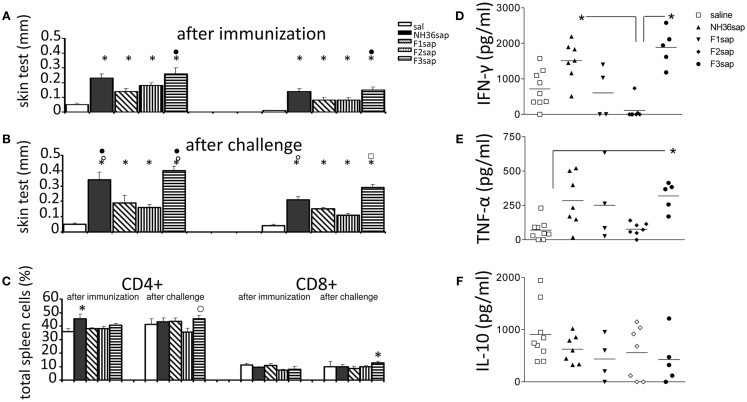
**Intradermal response to the leishmanial antigen, flow cytometry analysis, and ELISA of cytokines in supernatants of mice splenocytes**. IDR after immunization **(A)** and after challenge **(B)** 24 h (left) and 48 h (right) after antigen injection. Splenocytes after *in vitro* culture for 3 days at 37°C and 5% CO_2_ in the presence or absence of 5 μg/ml of recombinant NH36 and staining with anti-CD4 or anti-CD8 antibodies **(C)**. Results of two independent experiments with 9–10 mice per treatment group are shown as mean ± SE. *p < 0.05 from the saline controls, ● the F1, ○ the F2, or ⍁ from all the other vaccines. Secretions of IFN-γ **(D)**, TNF-α **(E)**, and IL-10 **(F)** in the supernatant of splenocytes, after challenge, are expressed in picogram per milliliters. Horizontal bars represent the mean values of one experiment (four to nine mice per treatment). *Significant differences between groups.

The cell-mediated immune response induced by immunization was initially assessed by the IDR to the *L. amazonensis* leishmanial antigen that was higher in all vaccinated animals than in controls prior to (Figure 2A) and after challenge (Figure 2B) (*p* < 0.0001 in both cases). After immunization, the F3sap vaccine induced higher footpad swelling than the F1sap vaccine. After challenge, the IDR responses were enhanced (*p* = 0.049 at 24 h and *p* = 0.007 at 48 h) mainly by the NH36sap, which was as potent as F3sap vaccine at 24 h after injection (Figure 2B). The preponderance of the F3sap vaccine was recovered 48 h after injection, when it induced the strongest intradermal reaction (Figure 2B). The proportions of anti-NH36-specific CD4+ and CD8+ lymphocytes in spleens were analyzed by FACS (Figure 2C). After immunization, the proportions of splenic CD4+ T cells of mice vaccinated with NH36 vaccine were higher than those of the saline controls. After challenge, and as expected for CL, the CD4+ proportions of saline control were sustained and only the F3 vaccine showed significantly increased proportions of NH36-specific CD4+ T cells over those of the F2 vaccine and of NH36-specific CD8+ T cells over the saline control (Figure 2C).

Six weeks after infection, the levels of cytokines were measured in supernatants of 10^6^ splenocytes after 3 days of *in vitro* culture with the addition of 5 μg of recombinant NH36. The results shown in Figures 2D–F are already subtracted from the values obtained without RPMI medium without antigen stimulation. Both the NH36sap (mean = 1510.15 pg/ml) and the F3sap-vaccinated mice (mean = 1888.85 pg/ml) showed higher concentrations of IFN-γ (*p* < 0.01 for both vaccines) than the F2sap-vaccinated mice (mean = 111.21 pg/ml) (Figure 2D). The TNF-α expression was increased only by the F3sap vaccine (mean = 318.87 pg/ml) over the saline controls (mean = 70.45 pg/ml) (*p* < 0.05) (Figure 2E) while no differences were detected in the IL-10 expression (Figure 2F). The secretion of IFN-γ and TNF-α was strongly correlated (*p* = 0.043). The levels of IFN-γ and TNF-α induced by the F1 vaccine did not achieve a significant difference compared to the F2 vaccine. However, the mean for IFN-γ (607.19 pg/ml) of the F1sap group fell outside the CI95% of the F2sap group (−221.17 to 332.39 pg/ml) (Figure 2D) and the mean for TNF-α of the F1sap group (370.28 pg/ml) also fell outside the CI95% of the F2sap group (77.44–77.52 pg/ml) (Figure 2E). No significant differences were observed between the levels of IL-10 generated by any treatment (Figure 2F). The supernatants represented in Figures 2D–F correspond to the lymphocytes, after challenge, represented in Figure 2C. At this point, lymphocytes represent 56.36% of the total splenocytes in culture (43.70% average of CD4 T lymphocytes + 12.66% average of CD8 T lymphocytes).

**Figure 3:**
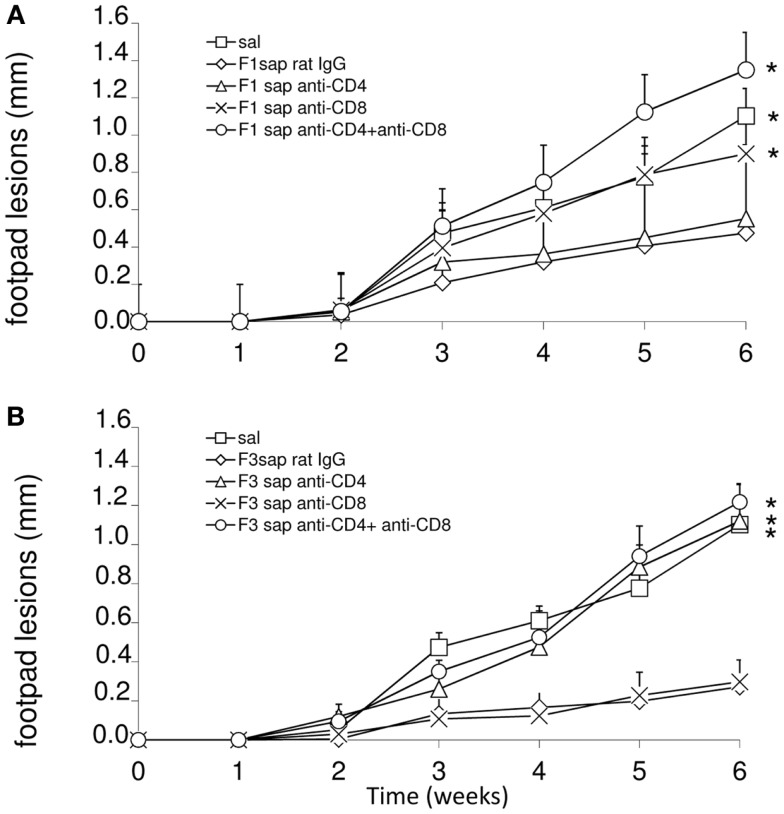
***In vivo* depletion assay with anti-CD4 and anti-CD8 monoclonal antibodies**. Mice were challenged with *L. amazonensis* after vaccination with F1sap **(A)** and F3sap **(B)** vaccines and treated with rat serum, anti-CD4+ or anti-CD8+, or the combination of anti-CD4 and anti-CD8 MAbs. Results are shown as the mean ± SD of the footpad measurements of one experiment (four to five animals per treatment) along the time. *p < 0.05, different from the F1sap **(A)** and F3sap **(B)** vaccines.

To detail the importance of CD4 and CD8+ epitopes of the F3 and F1 domains in cross-protection to *L. amazonensis* infection, we performed an *in vivo* depletion assay with anti-CD4 and anti-CD8+ monoclonal antibodies using mice immunized with F1sap and F3sap vaccines and challenged. The evolution of the sizes of footpad lesions is summarized in Figure 3. Significant differences among treatments were detected at week 6 (*p* < 0.0001). When compared to saline control, the F1sap vaccine determined a 57% (*p* = 0.008) reduction of footpad lesions that was not blocked by the anti-CD4-Mab (*p* = 0.413) but that was abolished by treatment with anti-CD8 antibody (*p* = 0.016) (Figure 3A). On the other hand, when compared to the saline control, the F3sap vaccine (Figure 3B) determined a 75% (*p* = 0.008) reduction in footpad lesion that was blocked by anti-CD4+ antibodies (*p* = 0.016 compared to the F3sap vaccine) but not impaired by depletion with anti-CD8-Mab (*p* = 0.730 compared to the F3sap vaccine). Our results indicate that the reduction of the size of lesion generated by F1sap vaccine is mainly mediated by CD8 epitopes present in the sequence of the F1 domain while reduction of lesion size induced by the F3sap vaccine is related to the presence of CD4+ epitopes in the F3 domain.

**Figure 4:**
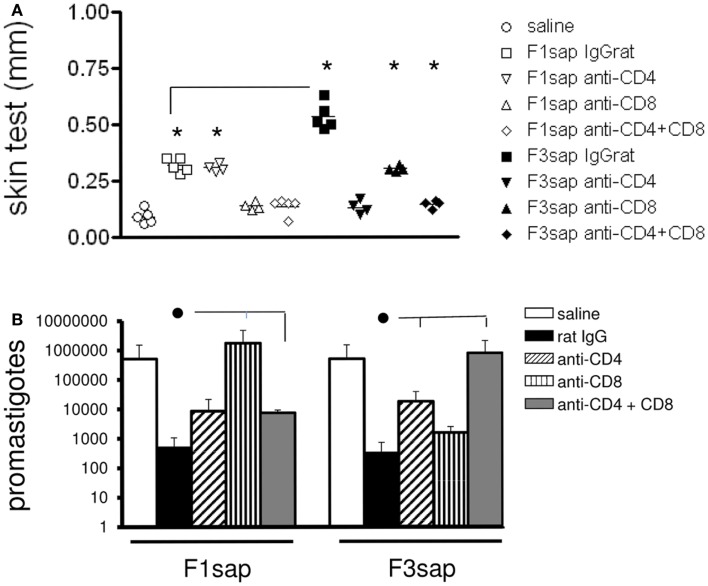
**Intradermal response and number of parasites in footpad lesions of mice submitted to an *in vivo* depletion assay with anti-CD4 and anti-CD8 monoclonal antibodies**. The IDR to *Leishmania amazonensis* lysate was measured in F1sap- and F3sap-vaccinated animals that were challenged with *L. amazonensis* and treated with rat serum, anti-CD4 or anti-CD8, or the combination of anti-CD4+ and anti-CD8+ MAbs (A). IDR was measured 6 weeks after challenge and 48 h after antigen injection. Results of one experiment with four to five mice per treatment group are shown. *p < 0.05, different from the saline controls and horizontal lines represent significant differences between the two vaccines **(A)**. In the limiting dilution assay **(B)** bars represent the number of promastigotes ± SD in each treatment (one experiment with four to five mice per treatment). ● Horizontal lines express significant differences from the F1sap- or F3sap-vaccinated treated with rat IgG only.

This hypothesis was also supported by the analysis of IDR after challenge, which disclosed significant differences among treatments (*p* < 0.0001) both at 24 h (not shown) and 48 h after antigen injection (Figure 4A). IDR was increased above the saline controls, in mice vaccinated with F1sap (*p* < 0.008), treated or not with anti-CD4+ Mab (*p* = 0.02), but it was decreased after treatment with anti-CD8+ and both anti-CD4 and -CD8 antibodies (Figure 4A) suggesting that the IDR response enhancement is related to epitopes for CD8+ T cells located in the F1 domain. The F3sap vaccine, showed a stronger IDR than the F1sap vaccine (*p* = 0.008) (Figure 4A), that was abolished by anti-CD4 Mab but not anti-CD8 Mab suggesting that it was mainly mediated by CD4+ T cells with a partial contribution of CD8+ T lymphocytes. The size of footpad lesions on week 6 showed significant negative correlation to the results of intradermal response (*R* = −0.79; *p* < 0.0001; *R*^2^ = 0.63 for IDR 24 h; and *R* = −0.82; *p* < 0.0001; *R*^2^ = 0.68 for IDR at 48 h) confirming that IDR is a good correlate of protection.

In correlation with these results, the parasite load in footpad lesion, evaluated by a limiting dilution assay (Figure 4B), also disclosed that protection induced by F1sap was abolished in mice treated with anti-CD8 Mab (*p* = 0.032) while protection generated by the F3sap vaccine was absent in mice treated with anti-CD4 Mab (*p* = 0.016). When compared to the saline controls (514,850 promastigotes), 99.93% (513 promastigotes) and 99.90% (341 promastigotes) reductions in the number of parasites were determined by the F3 and the F1 vaccines, respectively. The log_10_ values of parasite load in footpads correlated significantly with the increase in IDR (*R* = −0.6734; *p* < 0.0001; *R*^2^ = 0.4534) and with the decrease in footpad lesions (*R* = 0.5994; *p* < 0.0001; *R*^2^ = 0.3593) confirming that NH36 vaccine generated cross-protection against cutaneous leishmaniasis is determined by CD8 epitopes of F1 domain and by CD4 epitopes in the F3 domain.

The secretion of IFN-γ (*R* = −0.5518; *p* = 0.002; *R*^2^ = 0.3045) and TNF-α (*R* = −0.4655; *p* = 0.011; *R*^2^ = 0.2162) was negatively correlated with the increase of footpads lesions sizes (not shown) and thus, were strong correlates of protection against *L. amazonensis* infection.

The superiority of the F3 over the NH36 vaccine was evident in many variables. We calculated the increment in the immunoprotective effect of the F3 vaccine taking into consideration all the variables that showed significant differences between the two formulations (Table 1). We found that the F3 vaccine developed a 40.40% higher average protective effect than the NH36 vaccine.

**Table 1:** **Superiority of the F3 peptide domains over the NH36 vaccine in prophylaxis against *L. amazonensis* infection**.

Variable	F3	NH36	Enrichment (%)
IDR 48 h after challenge	0.290	0.210	27.58
INF-γ in supernatants	1888.85	1510.15	20.04
TNF-α in supernatants	322.47	284.95	11.64
Reduction of parasite load *L. amazonensis*	16.60	1.156	93.03
Mean + SD			40.40 + 27.77

*Calculation was performed according to the following equation = (F3 − NH36/F3) values × 100 = protective effect increment*.

**Figure 5:**
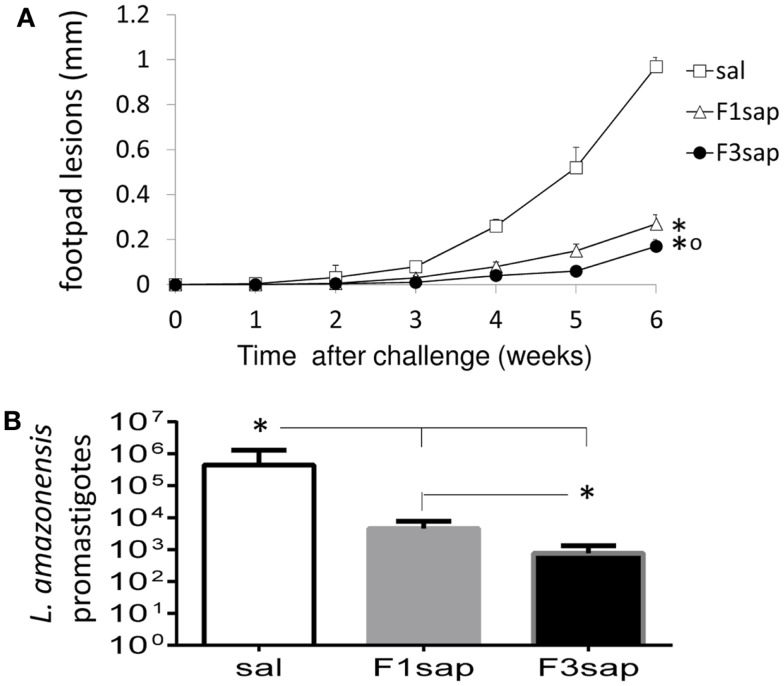
**Long-term cross-protection generated by the F3sap and F1sap vaccines**. Balb/c mice were vaccinated with three doses of F1sap or F3sap with a weekly interval and challenged with *L. amazonensis* infective promastigotes, 30 days after the last immunization. The evolution of the sizes of footpad lesions **(A)** and the parasite load in lesions (limiting dilution assay) **(B)** were determined. Bars represent the mean ± SD of one experiment with 10 mice for each treatment. *p < 0.05 significant differences from the saline controls and ∘ from the F1sap vaccine.

We further assessed the possible long-term cross-protection generated by the F3sap and F1sap vaccines in Balb/c mice that received three weekly interval vaccinations but that were challenged 1 month after the last vaccine dose. Significant reductions in the sizes of footpad lesions were achieved by vaccination with the F1sap (72%, *p* = 0.0003) and the F3sap vaccine (99.82%, *p* = 0.0002). Six weeks after challenge, the F3 vaccine reduced the lesions more than the F1 vaccine (*p* = 0.002) (Figure 5A). When compared to the saline controls (*p* < 0.01), the limiting dilution assay analysis disclosed also a 99.82% level of protection generated by the F3 vaccine (mean promastigotes = 757) followed by a 98.97% reduction (4531.25 promastigotes) due to the F1 vaccine (Figure 5B). Parasite reduction was more pronounced in the F3 than in the F1 vaccine treated mice (*p* < 0.01).

**Figure 6:**
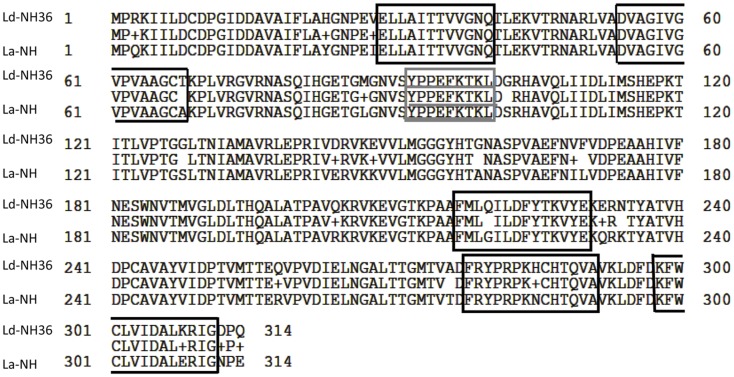
**Sequence analysis of nucleoside hydrolases of *Leishmania donovani* and *Leishmania amazonensis***. The sequences of the nucleoside hydrolases NH36 of *L. donovani* (Ld-NH36) and A34480 of *Leishmania amazonensis* (La-NH) were aligned using the BLASTP GenBank program. The line in the middle of the two sequences shows the amino acids share by the two NHs. The peptide sequence of MHC class II-IA^d^ and -IE^d^, haplotype H^2^ CD4+ T cell epitopes (34) are shown in black squares, on the F1 and F3 fragments. The amino acid sequence of MHC class I L^d^-CD8+ T cell predicted epitope of the F1 fragment (34) is underlined in the gray square.

The alignment of the amino acid sequences of *L. donovani* NH36 and the recently identified, NH A34480 of *L. amazonensis*, is represented in Figure 6. Both proteins are composed of 314 amino acids and show 93% of identity (292 from 314 amino acids) with no gaps. Additionally, we show the identity of the sequences of the predicted epitopes for CD4+ and CD8+ T cells, in the F1 and F3 domains of both proteins (Figure 6). The first epitope for CD4+ and the epitope for CD8+ T cells of the F1 domain of the two *Leishmanias* are conserved showing total identity, while the second epitope for CD4+ T cell shows a difference only in the last amino acid. Indeed, Alanine (A) is present in *L. amazonensis* NH instead of the final threonine (T) of NH36 of *L. donovani*. Furthermore, a difference in only one amino acid was found in the sequences of the three epitopes for CD4+ lymphocytes of the F3 domain. In the first CD4+ epitope, glutamine (Q) is exchanged for glycine (G), in the second epitope, histidine (H) is substituted by asparagine (N), and in the third epitope, lysine (K) is replaced by glutamic acid (G) (Figure 6).

## Discussion

We were able to disclose the antigenic basis of NH36 of *L. donovani* in cross-protection to infection by *L. amazonensis*. Our results show that the global increase of the humoral and cellular immune response promoted by the F3sap vaccine and the increase of the antibody response, IFN-γ and TNF-α secretion by the F1sap vaccine determined the vaccine protection against the *L. amazonensis* challenge. We also demonstrated that the cellular immune response induced by the F3 peptide (C-terminal domain) against the *L. amazonensis* infection is superior to the one induced by the cognate NH36 protein suggesting that it holds the main NH36 sequences responsible for the TH1 immune response. The increased IFN-γ and TNF-α secretion in supernatants confirmed the predominance of the immunogenicity of the F3 peptide. On the other hand, the F1 vaccine induced a weaker but significant DTH response and a mild enhancement of IFN-γ and TNF-α levels.

In a previous work, we demonstrated that protection against *L. chagasi* generated by the NH36 vaccine is related to its C-terminal domain and is mediated mainly by a CD4+ T cell-driven response with a lower contribution of CD8+ T cells (34). Increases in IgM, IgG2a, IgG1, and IgG2b antibodies, CD4+ T cell proportions, IFN-γ secretion, ratios of IFN-γ/IL-10 producing CD4+ and CD8+ T cells, and percents of antibody binding inhibition by synthetic predicted epitopes were detected in F3-vaccinated mice. The increases in DTH and in ratios of TNFα/IL-10 CD4+ producing cells were however the strong correlates of protection, which was confirmed by *in vivo* depletion with monoclonal antibodies, algorithm predicted CD4 and CD8 epitopes and a pronounced decrease in parasite load (90.5–88.23%; *p* = 0.011) that was long-lasting. No decrease in parasite load was detected after vaccination with the N-domain of NH36, in spite of the induction of IFN-γ/IL-10 expression by CD4+ T cells after challenge. Both peptides reduced the size of footpad lesions, but only the C-domain reduced the parasite load of mice challenged with *L. amazonensis* (34).

In the present study, as detected in the previous investigation (34), the antibody response also indicated the predominance of the F3 followed by the F1 peptide. This occurred, in the *L. amazonensis* model, mainly after challenge. In the *L. chagasi* model (34), the F3 was the only peptide to induce levels of IgG and IgG2a antibodies as high as those of the NH36 vaccine. After *L. chagasi* challenge, the IgG2a levels were 34% higher in the F3sap than in the F1sap vaccine group. In the *L. amazonensis* model, both F3 and F1 peptides seem to have similar degrees of contribution to the humoral response. Antibodies to the F1 peptide were also increased in infected dogs after immunotherapy with the NH36 DNA vaccine (33). Coincidentally, two B cell epitopes for dog and human antibodies were identified along the sequence of NH36 (37). Peptide 17 (TPAVQKRVKEVGTKP) (37) overlaps with the epitope that we previously identified in the sequence of F3 (*AVQKRVKEVGTKP*AAFML) (34), which was responsible for the highest inhibition of antibody binding to NH36 (31.40%). Peptide 18 (TTVVGNQTLEKVT) (37) overlaps with the single antibody epitope that we previously identified in the F1 fragment (*NQTLEKVT*RNARLVADVAG) (34). Peptide 17 developed 100% sensitivity and 100% specificity against sera of canine VL and 100% sensitivity human VL samples (37). All these results suggest that the NH36 B epitopes are good candidates for immunodiagnosis of both visceral and cutaneous leishmaniasis (33, 34, 37) and that the F3 and F1 are good candidate for a bivalent vaccine.

Regarding the results of vaccination against the challenge by *L. chagasi* (34) or *L. amazonensis*, the IDR response and the increase of the proportions of lymphocytes after *in vitro* culture with NH36 showed similarities. In both models, the F3 vaccine was imunodominant, meaning that the strong contribution to protection against cutaneous leishmaniasis by the F1 peptide is not revealed by these variables.

Common protective effects of the F3 vaccine against the infections by *L. chagasi* (34) and *L. amazonensis* also include the increase of: DTH response, TNF-α expression over that of IFN-γ, levels of CD4+ and CD8+ NH36-specific splenocytes, and the impairment of the protective efficacy by depletion of the CD4+ T cells (34), which indicate that cross-protection is mediated by a TH1 response induced against CD4+ epitopes of F3. This is an outstanding property of the C-terminal domain of NH36 considering the difficulties to obtain CD4+ mediated immune protection against protozoa infections (38). The F1 vaccine, on the other hand, did not reduce the *L. chagasi* parasite load, despite the induction of the IFN-γ/IL-10 expression by CD4+ T cells (34), but reduced 57–99% of footpad lesions and parasite load, respectively, in *L. amazonensis* infection and this decrease was impaired by treatment with anti-CD8+ Mab. CD8 T cells have proved to be important in infection clearance promoting localized restricted lesions and being absent in lesions of diffuse cutaneous leishmaniasis patients (39). Thus, the identification of an antigen promoting a CD8 T cell-driven protection is worthy.

The *in vivo* depletion assay with anti-CD4 and anti-CD8 monoclonal antibodies disclosed that protection against *L. chagasi* infection induced by the NH36sap vaccine involved the function of CD4 and CD8+ lymphocytes (34). The CD4 protection was mainly related to the epitopes of F3 (34). The lack of efficacy of F1sap vaccine, a strong inducer of a CD8 T cell response, against *L. chagasi* infection, is explained by the importance of CD4+ T cell response in the immunosuppressive characteristic of VL (34). Indeed a 22% decrease in the CD4+ T cell proportions was detected in mice infected with *L. chagasi* while conversely, the CD4+ levels remained stable after *L. amazonensis* infection. Our results revealed that while the participation of CD4+ T cells is responsible for the protection against *L. chagasi* infection (34), the combined function of CD8+ T and CD4+ T cells is necessary for vaccine efficacy against infection with *L. amazonensis*, and this will be probably achieved by using the two peptide domains in vaccination against cutaneous leishmaniasis.

Therefore, while the F3 peptide hosts the immunodominant CD4+ epitopes necessary for protection against *L. chagasi* and *L. amazonensis*, the F1 peptide exerts a co-dominance in immunoprotection to *L. amazonensis* infection, which is mediated mostly by CD8+ epitopes. Interestingly, a high affinity epitope for CD8+ T cells (YPPEFKTKL) was described in our previous work inside the sequence of the F1 peptide (34).

Immunization with the F3 peptide exceeded in 36.73% the protective response induced by the cognate NH36 protein against *L. chagasi* (34) and in 40.40% the protection induced against *L. amazonensis*. These results indicate that vaccine formulations including F3 might show the best results against visceral leishmaniasis while a combination of F3 and F1, or a potential chimera might be needed for protection against both visceral and cutaneous leishmaniasis.

Our results also demonstrate the induction of long-term cross-protection by the F3 followed by the F1 vaccine. Indeed, strong reduction of lesion size and parasite load reduction were detected in mice challenged 1 month after vaccination suggesting that both vaccines are able to generate both effector and memory T cells responsible for the immunoprotective response.

Despite the many antigens tested for vaccination in laboratory models (7, 8) only a few are under analysis as tentative synthetic vaccines against *Leishmania* (40–44). The kmp-11 (40) and the amastigote A2 (43) contain units encoding CD8+ cytotoxic T lymphocyte epitopes while the polyprotein Leish110f (8, 41), the LACK158–173 peptide (42), the amastigote A2 antigen (43), and the MML-triple fusion *L. major* vaccine expressed in Adenovirus (44) trigger a Th1-biased CD4+ T cell response.

Since the NH36 function is mandatory at the early stages of the parasite infection and is a strong phylogenetic marker (24, 25) with significant homology to the sequences of NH of *L. major* (95%) (25), *L. chagasi* (99%)*, Leishmania infantum* (99%), *Leishmania tropica* (97%), *Leishmania mexicana* (93%), *Leishmania braziliensis* (84%) (26), the achievement of high protection using the *L. donovani* NH36 vaccine against the challenge by *L. chagasi* was expected (34). The previous finding of cross-protection against *L. mexicana* induced by vaccination with NH36 supported this premise (29). Recently, the genome sequence of *L. amazonensis* was described (35) and the presence of the gene of NH A34480 was disclosed. We describe here that this gene shows 93% of identity to the sequence of NH36 of *L. donovani*. Additionally, we detected that the epitope for CD8+ T cells (34), and one epitope for CD4+ T cells of the F1 domain are completely conserved in *L. amazonensis* NH, while the other CD4+ epitopes of the F1 and F3 domains differ in a single amino acid, having the rest of their sequences preserved. These results reveal the structural basis of the demonstrated cross-immune protection induced by the *L. donovani* F1 and F3 vaccines in prophylaxis to the infection by *L. amazonensis*, and encourage us to pursue the development of a T cell epitope synthetic bivalent vaccine for prophylaxis against both leishmaniasis. The C-terminal and the N-terminal domains of NH36 could be potentially combined into a chimera, for the bivalent vaccine. Since NH of *L. donovani* also shares 68% identity with *Haemophilus influenzae* and 30% identity and conserved motifs with *Bacillus anthracis* (45) and NHs are also found in yeasts (46) and insect cells (47), the identification of shared NHs domains might allow the rational design development of cross-protective subunit or synthetic vaccines for protection against multiple purine salvation pathway-dependent pathogens.

To our knowledge, this is the first case of a second-generation licensed vaccine to evolve DNA to a recombinant defined protein formulation that might be used in a potential bivalent vaccine against cutaneous and visceral leishmaniasis.

## Author Contributions

Conceived and designed the experiments: Clarisa B. Palatnik-de-Sousa, Dirlei Nico. Acquisition, analysis, and interpretation of data: Dirlei Nico, Daniele Crespo Gomes, Marcus Vinícius Alves-Silva, Elisangela Oliveira Freitas, Alexandre Morrot, Diana Bahia, Clarisa B. Palatnik-de-Sousa, Marcos Palatnik, Mauricio M. Rodrigues. Wrote the paper: Clarisa B. Palatnik-de-Sousa. Final approval of the last version of the manuscript to be published: Clarisa B. Palatnik-de-Sousa, Dirlei Nico, Daniele Crespo Gomes, Marcus Vinícius Alves-Silva, Elisangela Oliveira Freitas, Alexandre Morrot, Diana Bahia, Marcos Palatnik, Mauricio M. Rodrigues.

## Conflict of Interest Statement

The authors have declared that there is no competing interest. This work is related to the pendent Patent PI1015788-3, INPI, Brazil.
